# Supplementary material for: Glucose and Oxygen Metabolism Coordinate Human Cortical Developmental Decisions
Source: bioRxiv. 2025 Dec 5:2025.12.02.691634. Preprint. [Version 1] doi: 10.64898/2025.12.02.691634 (PMC12822575; doi:10.64898/2025.12.02.691634)
Supplement: Supplementary file 1 [file NIHPP2025.12.02.691634V1-supplement-1.pdf]

bioRxiv preprint doi: https://doi.org/10.64898/2025.12.02.691634; this version posted December 5, 2025. The copyright holder for this preprint (which was not certified by peer review) is the author/funder, who has granted bioRxiv a license to display the preprint in perpetuity. It is made available under a CC-BY 4.0 International license.

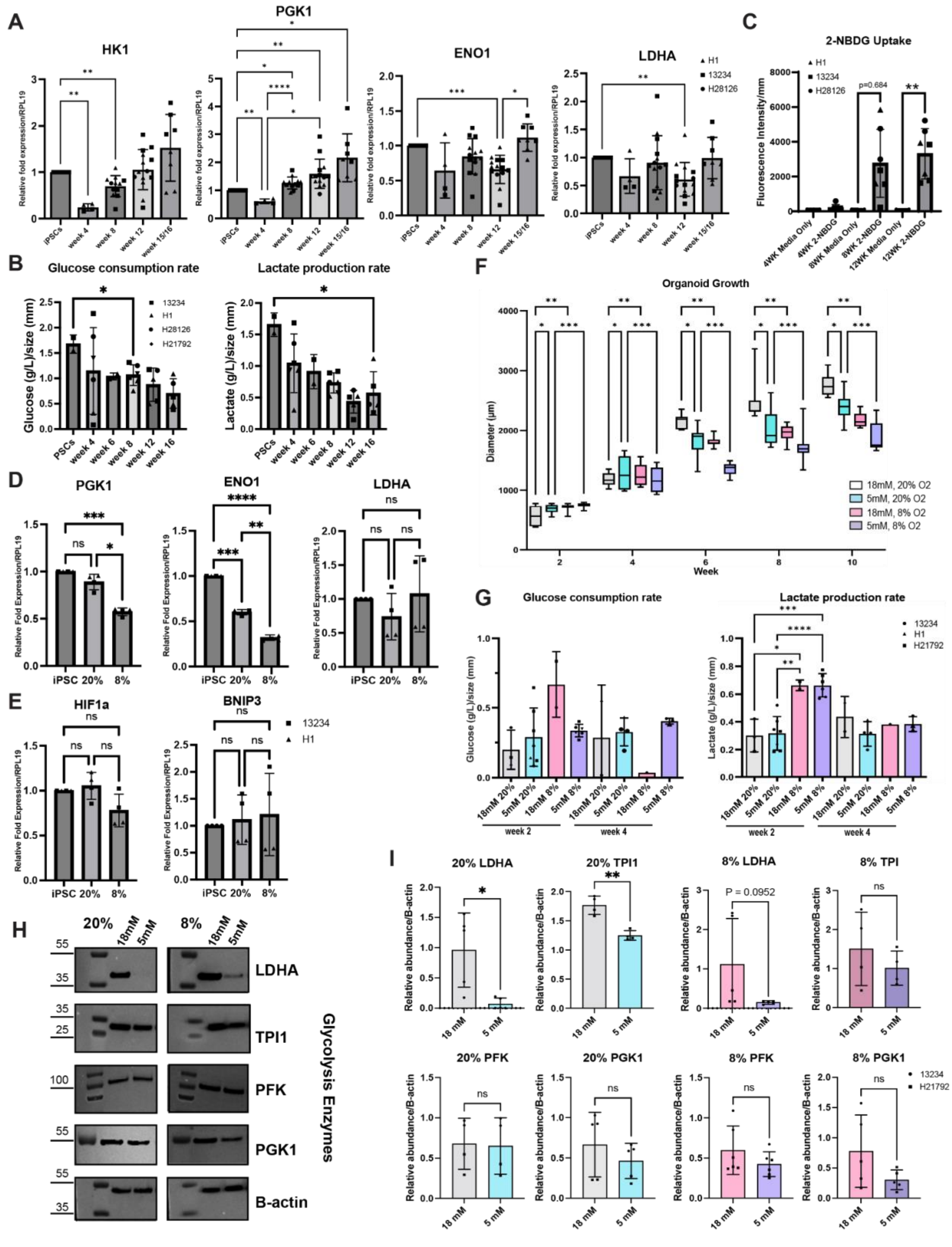

S Figure 1: Organoids cultured under standard conditions have unclear metabolic

bioRxiv preprint doi: https://doi.org/10.64898/2025.12.02.691634; this version posted December 5, 2025. The copyright holder for this preprint (which was not certified by peer review) is the author/funder, who has granted bioRxiv a license to display the preprint in perpetuity. It is made available under a CC-BY 4.0 International license.

transitions.

A) q-RT-PCR on glycolysis genes was performed on iPSCs and organoids from the same lines until 16 weeks of differentiation (HK1: One-way ANOVA, iPSC vs wk4: **p<0.0013, iPSC vs wk8: **p<0.0058; ENO1: One-way ANOVA, iPSC vs wk12: ***p<0.0002, wk12 vs wk15/16: *p<0.0154; LDHA: iPSCs vs wk12: **p<0.0025; PGK1: iPSC vs wk4: **p<0.0097, iPSC vs wk8: *p<0.0112, iPSC vs wk12: **p<0.0072, iPSC vs wk15/16: *p<0.0366; wk4 vs wk8: ****p<0.0001; wk4 vs wk12: *p<0.0279). n=4 organoids pooled per differentiation batch from n=3 iPSC lines are collected for q-RT-PCR repeated n=2 times each per line. Data are mean ± SD.

B) Spent media analysis. Glucose consumption minimally changed over time under baseline culture conditions (Ordinary one-way ANOVA with Tukey's multiple comparisons, PSC vs wk8: *p<0.0211). Lactate production gradually decreased without significant change until gliogenic periods (Ordinary one-way ANOVA with Tukey's multiple comparisons, PSC vs wk16: *0.0211). Spent media collected from organoids from n=4 lines across time points. Data are represented as mean ± SD.

C) Uptake of 2-NBDG in organoids increases during neurogenic and gliogenic stages (Ordinary one-way ANOVA, Wk8 media vs Wk8 2-NBDG: p=0.0684; Wk12 media vs Wk12: **p<0.0069). n=3 organoids per differentiation batch each from n=3 iPSC lines. Data are represented as mean ± SD.

D) q-RT-PCR was performed in organoids cultured under 20 vs 8% O_2 conditions. Under 8% conditions, expression of glycolysis genes *PGK1* and *ENO1* decreased (Ordinary one-way ANOVA with Tukey's multiple comparisons, PGK1: iPSCs vs 8% O_2 : ***p<0.0005; 20% vs 8% O_2: *p<0.0101; ENO1: iPSCs vs 20% O_2 : ***p<0.0002; iPSCs vs 8% O_2 : ****p<0.0001; 20% vs 8% O_2: **p<0.0032). n=4 organoids pooled per differentiation batch from n=3 iPSC lines are collected for q-RT-PCR repeated n=2 times per line. Data are represented as mean ± SD.

E) q-RT-PCR was performed on organoids cultured under 20 vs 8% O_2 conditions. Under 8% conditions, expression of hypoxia genes *HIF1a* and *BNIP3* did not change. n=4 organoids pooled per differentiation batch from n=3 iPSC lines are collected for q-RT-PCR and repeated n=2 times each per line. Data are represented as mean ± SD.

F) Organoid size changes over time and under different conditions (Two-way ANOVA with multiple comparisons: 18mM 20% vs 5mM 20%: *p=0.0229, 18mM 20% vs 18mM 8%:**p=0.0012, 5mM 20% vs 5mM 8%: ***p=0.0001, n>7 organoids/timepoint from n=3 PSC lines). Data are represented as mean ± SD.

G) Glucose consumption per conditions for weeks 2-4 shows most significant increases in 8% O_2 conditions (Ordinary one-way ANOVA with Tukey's multiple comparisons, n>3 spent media collections per timepoint from n=3 lines). Lactate production for week 2-4 with most significant increase in 8% O_2 conditions at week 2 (Ordinary one-way ANOVA with Tukey's multiple comparisons: Week 2: 18mM 20% vs 18mM 8%: *p<0.0267; 18mM 20% vs 5mM 8%: ***p<0.0006; 5mM 20% vs 18mM 8%: **p<0.0093; 5mM 20% vs 5mM 8%: ****p<0.0001; n>3 spent media collections per timepoint from n=3 lines). Datapoints are metabolite concentration values/line ± SD.

H-I) Western blot analysis of glucose metabolism enzymes. Under 20% O_2 conditions, LDHA and TPI1 decreased after treatment with 5mM glucose, but there were no significant changes in other glucose metabolism proteins. (20% LDHA: Shapiro-Wilk normality test: *p<0.027, Mann-Whitney test: *p<0.0159; 20% TPI1: Shapiro-Wilk normality test: p=0.143, unpaired t test: **p<0.0011).

bioRxiv preprint doi: https://doi.org/10.64898/2025.12.02.691634; this version posted December 5, 2025. The copyright holder for this preprint (which was not certified by peer review) is the author/funder, who has granted bioRxiv a license to display the preprint in perpetuity. It is made available under a CC-BY 4.0 International license.

n=4 organoids pooled per differentiation batch from n=2 iPSC lines are collected for western blot and repeated n=2 times each per line. Data are represented as mean ± SD.

bioRxiv preprint doi: https://doi.org/10.64898/2025.12.02.691634; this version posted December 5, 2025. The copyright holder for this preprint (which was not certified by peer review) is the author/funder, who has granted bioRxiv a license to display the preprint in perpetuity. It is made available under a CC-BY 4.0 International license.

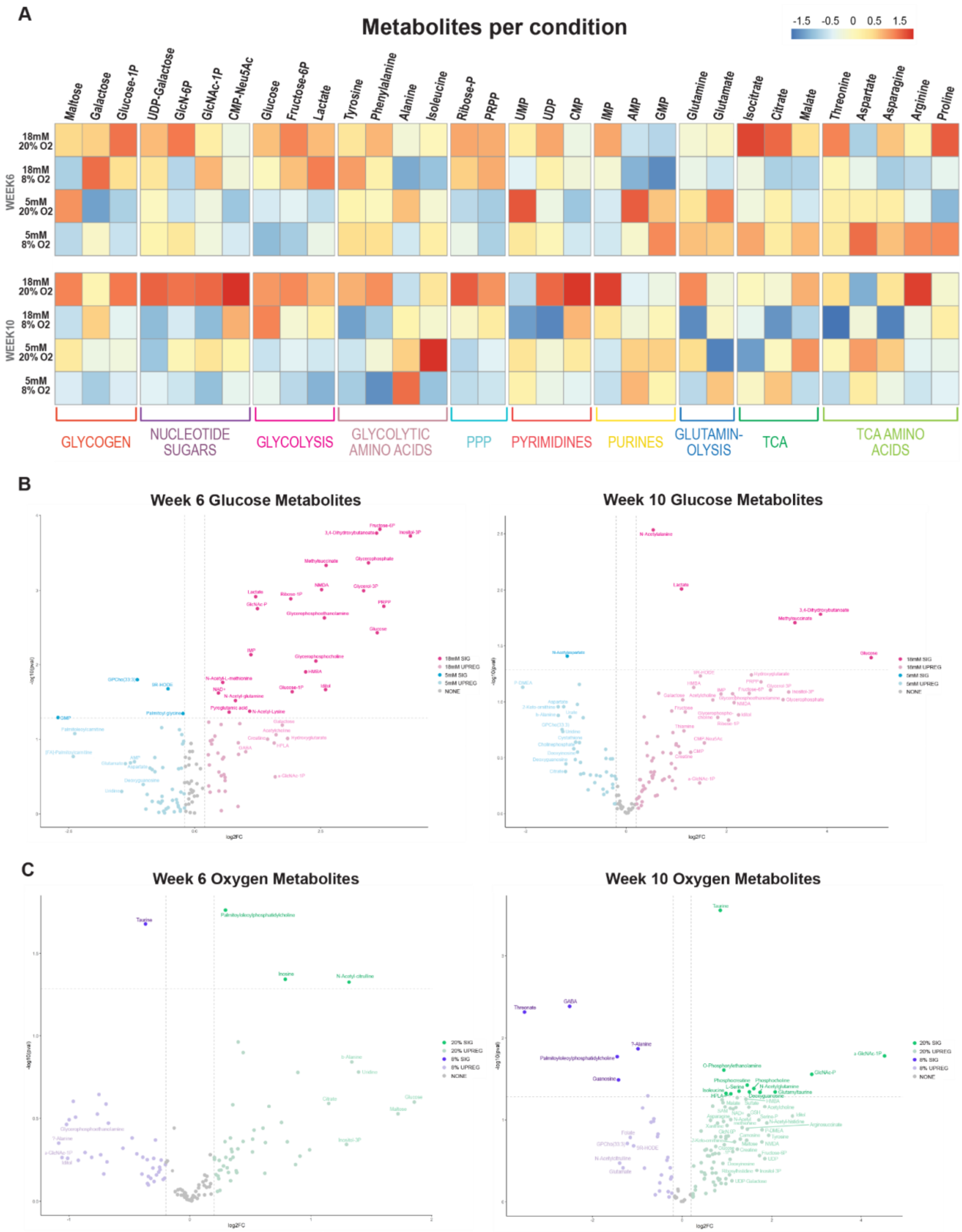

S Figure 2: Glucose and oxygen conditions impact metabolite abundance.

bioRxiv preprint doi: https://doi.org/10.64898/2025.12.02.691634; this version posted December 5, 2025. The copyright holder for this preprint (which was not certified by peer review) is the author/funder, who has granted bioRxiv a license to display the preprint in perpetuity. It is made available under a CC-BY 4.0 International license.

A) Heatmap summarizes changes in metabolite composition per condition across time. Metabolites were extracted from n=5 organoids pooled per differentiation batch from n=3 iPSC lines/condition.

B) 18mM glucose conditions had more metabolites significantly upregulated compared to 5mM glucose, particularly glycolysis regulators, such as glucose, lactate, and Fructose-6P, at both 6 and 10 weeks of differentiation.

C) Few metabolites differed between O_2 conditions at week 6, but at week 10, 8% O_2 had increase in GABA, Threonate, L-Alanine and 20% O_2 had increased Taurin, GlcNAc-P, O-Phosphorylethanolamine. n=5 organoids pooled per differentiation batch from n=3 iPSC lines/condition were collected for metabolite extraction.

bioRxiv preprint doi: https://doi.org/10.64898/2025.12.02.691634; this version posted December 5, 2025. The copyright holder for this preprint (which was not certified by peer review) is the author/funder, who has granted bioRxiv a license to display the preprint in perpetuity. It is made available under a CC-BY 4.0 International license.

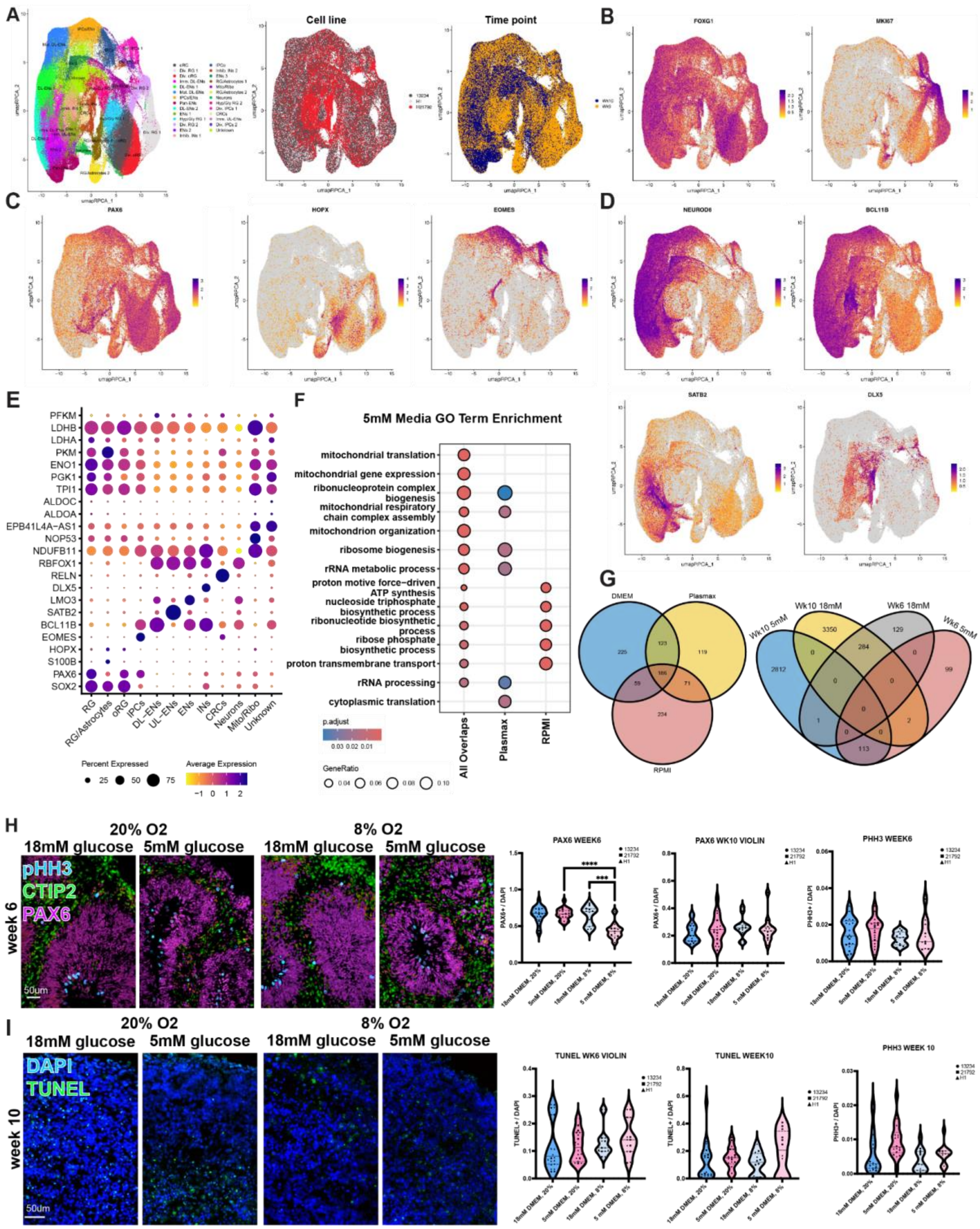

S Figure 3: Physiologic glucose and oxygen conditions have minimal impact on proliferation, lineage shifts, & cell death in organoids.

A) UMAPs of collected organoid data by more granular cluster annotation, iPSC line, and timepoint of collection.

bioRxiv preprint doi: https://doi.org/10.64898/2025.12.02.691634; this version posted December 5, 2025. The copyright holder for this preprint (which was not certified by peer review) is the author/funder, who has granted bioRxiv a license to display the preprint in perpetuity. It is made available under a CC-BY 4.0 International license.

B-D) Feature plots of cortical (*FOXG1*), progenitor (*KI67, PAX6, HOPX, EOMES*), and neuronal marker (*NEUROD6, BCL11B, SATB2, DLX5*) genes.

E) Dotplot indicates marker expression and percent of cells each gene is expressed across clusters. Canonical cell type marker genes, top DEGs for each cell type, and glycolysis genes are shown.

F) GO term enrichment dotplot of significantly enriched terms found in all overlapped 5mM media DEGs from all cells across timepoints (DMEM/F12, Plasmax, RPMI) compared against the unique DEGs to each media (Plasmax, RPMI). DMEM/F12 had no significantly enriched terms.

G) Venn diagrams showing the overlapping DEGs from all cells regardless of timepoint between medias (DMEM/F12, Plasmax, RPMI; left diagram) and the DEGs from all cells across glucose concentration (18mM , 5mM) and timepoint (wk6, wk10) (right diagram).

H) Organoids cultured under different glucose and oxygen conditions had minimal impact on PAX6+ RG without impacting pHH3+ proliferation. At week 6 there was a small decrease at week 6 under 8% O_2 conditions (One way ANOVA, PAX6 week6: 5mM 20% vs 5mM 8%: ****p<0.0001, 18mM 8% vs 5mM 8%: ***p<0.0001).

I) Organoids cultured under different glucose and oxygen conditions did not increase the number TUNEL+ dying cells. n=4 organoids per differentiation batch each from n=3 iPSC lines/condition/timepoint. Data are represented as mean ± SD.

bioRxiv preprint doi: https://doi.org/10.64898/2025.12.02.691634; this version posted December 5, 2025. The copyright holder for this preprint (which was not certified by peer review) is the author/funder, who has granted bioRxiv a license to display the preprint in perpetuity. It is made available under a CC-BY 4.0 International license.

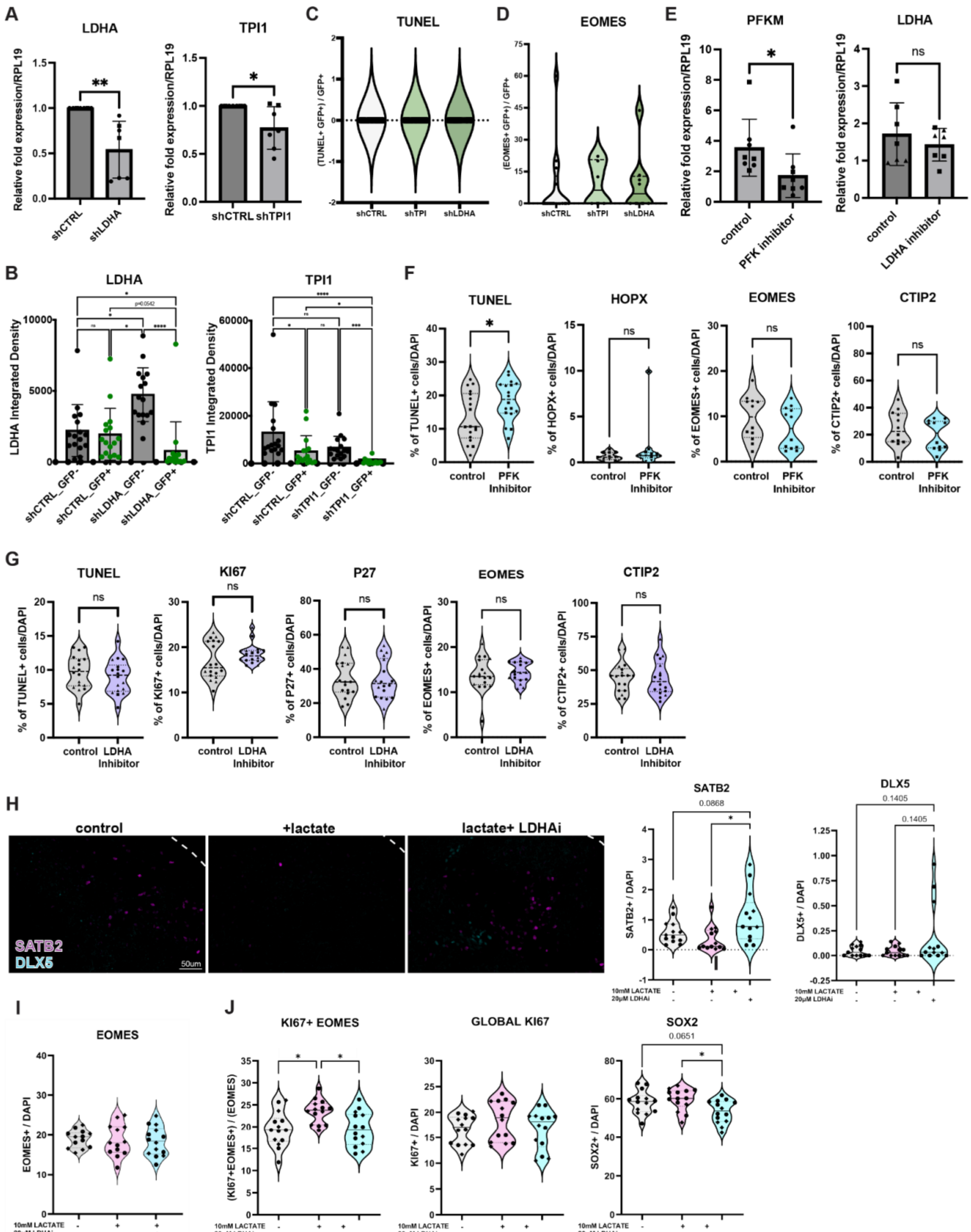

S Figure 4: Changes in glycolysis have cell-specific impact during neurodevelopment
A) Electroporation of LDHA and TPI1 shRNAs decreased levels of respective RNA expression by q-RT-PCR (LDHA: Welch's t-test: **p<0.0083; TPI1: Welch's t-test: *p<0.0337).

bioRxiv preprint doi: https://doi.org/10.64898/2025.12.02.691634; this version posted December 5, 2025. The copyright holder for this preprint (which was not certified by peer review) is the author/funder, who has granted bioRxiv a license to display the preprint in perpetuity. It is made available under a CC-BY 4.0 International license.

B) Electroporation of LDHA and TPI1 shRNAs result in decreased abundance of targeted protein in electroporated GFP+ cells with knockdown (One way ANOVA with multiple comparisons, shcontrol GFP+ vs shLDHA GFP+: p=0.0542; shLDHA GFP- vs shLDHA GFP+: p<0.0001; shcontrol GFP- vs shLDHA GFP+: *p<0.0157; shcontrol GFP+ vs shTPI1 GFP+: *p<0.0397; shTPI1 GFP- vs shTPI1 GFP+: ***p<0.0001).

C-D) There are no significant changes in TUNEL+ or EOMES+ cells after electroporation of shTP1 or LDHA (One way ANOVA, EOMES: shTPI1: p=0.9885; shLDHA: 0.9672).

E) Week 6 organoids treated with PFK inhibitor, PFK15, had decreased relative PFK expression via q-RT-PCR compared to controls (Unpaired student's t-test: *p<0.0464). Treatment with LDHA inhibitor, GSK2837808A, did not impact LDHA relative expression (Unpaired student's t-test: p=0.676)

F) PFK inhibition resulted in a small increase in TUNEL+ dying (Unpaired student's t-test: *p<0.0282), without change to HOPX+ oRG (Shapiro-Wilk normality test: p<0.0001; Mann-Whitney test: p=0.3186), EOMES+ IPC (unpaired t test: p=0.2621) or CTIP2+ DL-EN (unpaired t test: p=0.3612).

G) LDHA inhibition did not impact number TUNEL+ dying (unpaired t test: p=0.3752), KI67+ proliferating cells (Shapiro-Wilk normality test: p=0.0134; Mann-Whitney test: p=0.161), p27+ differentiating cells (unpaired t test: p=0.8708), EOMES+ IPCs (unpaired t test, p=0.5612), or CTIP2+ DL-EN (unpaired t test, p=0.8819). n=4 organoids per differentiation batch each from n=3 iPSC lines/condition.

H) Lactate alone did not significantly affect the proportion of SATB2+ UL-ENs (control vs lactate: p= 0.3220) or DLX5+ IN (control vs lactate: p= 0.1502) at week 6, while the presence of LDHAi led to a significant increase in SATB2+ UL-ENs (lactate vs LDHA inhibitor: *p= 0.0116, control vs lactate+LDHAi: p= 0.0868) and trending increase in DLX5+ interneurons (lactate vs LDHA inhibitor: p = 0.1405, control vs lactate+LDHAi: p= 0.1405).

I) Lactate with and without LDHAi had no impact on EOMES+ cells (One way ANOVA: lactate: p=0.9542, lactate+LDHAi: p=0.9590).

J) Lactate significantly increased KI67+EOMES+ IPCs, which normalized in the presence of LDHAi (control vs lactate: *p=0.0145; lactate vs LDHA inhibitor: *p=0.0145), but did not impact global KI67+ cells (control vs lactate: p=0.4998 ; lactate vs LDHA inhibitor: p=0.4998, control vs lactate+LDHAi: p=0.9610). The proportion of SOX2+ cells was unaffected by lactate but significantly decreased with addition of LDHAi (control vs lactate: p=0.5497; lactate vs LDHA inhibitor: *p=0.0228, control vs lactate+LDHAi: p=0.0651).
